# Protocol parameter extraction and centralization framework for comprehensive and in‐depth CT protocol review and management

**DOI:** 10.1002/acm2.14316

**Published:** 2024-03-11

**Authors:** Andy LaBella, Da Zhang

**Affiliations:** ^1^ Department of Radiology Stony Brook University Stony Brook New York USA; ^2^ Department of Radiology Boston Children's Hospital Harvard Medical School Boston Massachusetts USA

**Keywords:** CT, protocol management, protocol review

## Abstract

CT protocol management is an arduous task that requires expertise from a variety of radiology professionals, including technologists, radiologists, radiology IT professionals, and medical physicists. Each CT vendor has unique, proprietary protocol file structures, some of which may vary by scanner model, making it difficult to develop a universal framework for distilling technical parameters to a human‐readable file format. An ideal solution for CT protocol management is to minimize the work required for parameter extraction by introducing a data format into the workflow that is universal to all CT scanners.

In this paper, we report a framework for CT protocol management that converts raw protocol files to an intermediary format before outputting them in a human‐readable format for a variety of practical clinical applications, including routine protocol review, protocol version tracking, and cross‐protocol comparisons.

The framework was developed in Python 3. Technical parameters of interest were determined via collaborative effort between medical physicists and lead technologists. Protocol files were extracted and analyzed from a variety of scanners across our hospital‐wide CT fleet, including various systems from Siemens and GE. Protocols were subcategorized based on relevant technical parameters into regular, dual‐energy, and cardiac CT protocols. Backend code for technical parameter extraction from raw protocol files to a JavaScript Object Notation (JSON) format was performed on a per‐system basis. Conversion from JSON to a readable output format (MS Excel) was performed identically for all scanners using the universal framework developed and presented in this work. Example results for Siemens and GE scanners are shown, including side‐by‐side comparisons for protocols with similar clinical indications.

In conclusion, our CT protocol management framework may be deployed on any CT system to improve clinical efficiency in protocol review and upkeep.

## 1 INTRODUCTION

Protocol management in CT refers to the organization and maintenance of routine CT protocols, which helps to achieve consistency in practice across all patients with similar demographics and clinical indications.^1^, ^2^ As CT systems have become less dependent on user‐configured parameters through the advent of automatic exposure control (AEC),^3^ hospitals have become more reliant on building large databases of CT protocols with dedicated, fined‐tuned settings for each clinical indication; thus, the practice of CT protocol management has become more arduous due to heavier burden on information systems, quality assurance and the process of protocol review.^4^ Along these same lines, manufacturers also have varying methods of deploying and describing their scanning (such as AEC) and reconstruction parameters, making it difficult to standardize CT protocols across different scanners.^3^, ^5^

Several solutions have been proposed to simplify modern CT protocol management, including using a master protocol system in which many branching protocols refer to a subset of overarching protocols.^4^, ^6^ Similarly, some institutions use standardized protocol templates to streamline the protocol review process,^7^ while others rely on more data‐driven approaches via the use of in‐house software.^8^, ^9^ Wiki‐based CT protocol webpages have been developed by institutions in order to have a centralized, version‐controlled database built upon an intuitive user‐interface.^10^, ^11^ However, master protocol systems require high upkeep and front‐end work to deploy, making them more suitable for healthcare facilities with a homogenous CT scanner fleet and less suitable for individual sites and small hospital networks, which may not have access to dedicated radiology IT professionals or imaging physicists.^12^ Small hospitals often don't have access to the large team of professionals required for protocol database development and maintenance, including dedicated medical physicists, protocol managers, and education specialists.^13^, ^14^ Commercial solutions for streamlined protocol management exist, such as GE Imaging Protocol Manager, Canon CT Vitality, and Siemens teamplay Protocols. While these software often come with remote access capabilities and user‐friendly interfaces, they may require service and are not vendor neutral, making them impractical and expensive for multi‐vendor sites since software would need to be purchased and maintained for each vendor. In addition, as new CT technologies are commercially deployed, such as iterative reconstruction^15^, ^16^ and photon‐counting CT,^17^ the technical parameters of interest for protocols will change, thus potentially requiring partial or complete overhauls of the entire protocol database.

As a result, a more practical solution for CT protocol management at any site is to maintain protocols on a per‐scanner basis, regardless of the size and scope of the facility's practice. On the technical implementation side, physicists should deploy a methodology that is customizable and can readily translate across different scanners, including ones that they may not have encountered before, to increase their flexibility and minimize the amount of front‐end effort required to parse out protocol parameters. To this end, we've developed a methodology for parsing technical parameters of interest from raw CT protocol files into an intermediate file format that is both intuitive and readily mutable (JavaScript Object Notation (JSON)) before outputting them in a human‐readable format for routine protocol review.

## 2 MATERIALS AND METHODS

### 2.1 Raw protocol file to JSON

We developed and deployed our methodology using Python 3.9 on three different models of CT scanners from two different vendors: Siemens Force, Siemens Intevo Bold (SPECT/CT), and GE Optima 660. Raw protocol files were exported directly from each scanner by medical physicists. Note that while this process may be directly handled by end‐users, depending on the level of knowledge and access provided by vendors on each CT scanner to end‐users, protocol file extraction may be better handled by technical experts from the vendors, such as field service engineers. Among these scanners, three different raw protocol file formats were found: XML for the Siemens Force, a general markup language for Siemens Intevo Bold, and “.proto” file for GE. XML is a prominent IT file format with a breadth of open‐source toolboxes and support, while “.proto” file is also a well‐known file format in the CT community that GE has used across several generations of CT scanners.^14^ In all protocol files, parameters are denoted by attribute‐ or tag‐ value pairs.^18^, ^19^, ^20^ Table 1 shows the different tags used to identify unique series in each of the 3 different protocol file formats, and how the parameters are organized and tagged in each series. Conversion from raw protocol files to JSON format was executed differently for each of the 3 file formats. This methodology was deployed on each of the technical parameters relevant to routine protocol review and optimization, which varied based on vendor, scanner, and protocol type.^21^, ^22^ The exact parameters to be extracted were also based on the protocol type, which was subdivided into regular, dual‐energy, and cardiac scans, based on the unique technical parameters for each.

**TABLE 1:** Protocol file format descriptions, including how scan and reconstruction series are tagged and uniquely identified, as well as how parameters are tagged and extracted from the raw protocol file to JSON format. Exact language taken from the protocol files are shown in quotations. Indentations or lack thereof reflect the syntax shown in the protocol files as well. Each of the three protocol file formats in this study has unique formats. Siemens XML and GE. proto files both have a tree‐like structure for identifying series, scans, reconstructions, and individual parameters along with their values. Siemens XML has unique tags, denoted as entry numbers (“EntryNo”), to delineate each major scan/recon within the protocol file, whereas general markup language and GE. proto rely on sequential listing of each series step without including an explicit unique identifier for each scan and recon.

	Siemens force (.xml)	Siemens SPECT/CT (Markup Language)	GE optima 660 (.proto)
Unique series identifier	“<MlModeEntryType …>” … “</MlModeEntryType …>”	“PROTOCOL_ENTRY_NO” … “MlModeEntry_Begin” … “MlModeEntry_End”	“Series” {…}
Scan identifier	“<MlModeEntryType …>” … “<**MlModeScanType** …>”	“PROTOCOL_ENTRY_NO” … “**MlModeScan_Begin**”	“Series” {… “**Group”** {…} }
Recon identifier	“<MlModeEntryType …>” … “<**MlModeReconType** …>”	“PROTOCOL_ENTRY_NO” … “**MlModeRecon_Begin**”	“Series” {… “**Group**” {… “Recon” {…} }}
Order identifier	“<MlModeEntryType **EntryNo** …>”	**N/A**—Sequential	**N/A**—Sequential
Individual parameter tag	**Scan** or **Recon** Identifier → <**tag**> value </**tag**>	**Scan** or **Recon** Identifier → **tag**: value	**Scan** or **Recon** Identifier → **tag** = value
Parameter extraction method	XML Element Tree	Manual	Manual

XML files were systematically parsed using the ElementTree Python XML parser.^23^ Each technical element of interest was identified based on its unique XPath.^24^ Each scan and reconstruction is given a unique entry number as a predicate, and the series and reconstruction descriptions are provided in a particular node in the corresponding XPath, thus enabling series identification without any prior knowledge about the protocol.^24^ As a result, every series can be identified blindly by iterating through the XML protocol file, and every parameter can be extracted and assigned to its corresponding series by calling on the proper node. Parameters were extracted based on the series type, which was subdivided into localizer (also referred to as “scout” or “topogram”), monitoring, premonitoring, and main scans. For cardiac scans, an additional series type in the protocol files denoted “bolus” was identified, which contained the parameters for the bolus tracking in contrast CT, including the timing and trigger levels.

Protocol files from the Siemens Intevo Bold scanner had an XML‐type structure but without many of the defining characteristics of XML, namely the axes (i.e., relative position) and unique paths for each node^24^; thus, we identified the file structure as a general markup language (extensions “.MlAdult” and “.MlChild”), and analyzed the files using an iterative structure: after identifying the relevant parameter names in the protocol files, we parsed through the files sequentially to parse out each series and reconstruction. A similar methodology to the one used for the Siemens Intevo Bold protocol files was deployed on the GE. proto files, which do not have unique identifiers for each scan and its technical parameters either.

### 2.2 JSON to human‐readable file

All chosen protocol file parameters were extracted and converted to JSON format.^25^ These JSON files are much more sparse and readable compared to the raw protocol files, making them suitable as an intermediate file format for conversion to the final, user‐friendly output format. Look‐up tables were generated for each protocol file type to extract protocol file parameters independently of the backend code. As a result, the JSON parameter files may be edited without changing the logic behind converting them from raw protocol files and converting to human‐readable files, respectively. Thus, the extracted parameters can be modified at any time with negligible changes to the front‐end. The primary changes that would need to be made are on the back‐end code when dealing with new scanners, series types, or protocol file formats. Regardless, converting to JSON first before human‐readable format compartmentalizes this process to make it more streamlined, manageable, and easier to debug. One example use case is when extracting tube settings from a conventional CT scan versus a dual‐energy scan, which would have 2 kVp settings instead of 1. The only modification necessary to extract the second kVp setting is to edit the JSON look‐up table, which would be modified to extract 2 unique kVp tag‐value pairs from the protocol file per main scan, whereas the backend code would stay exactly same.

**FIGURE 1:**
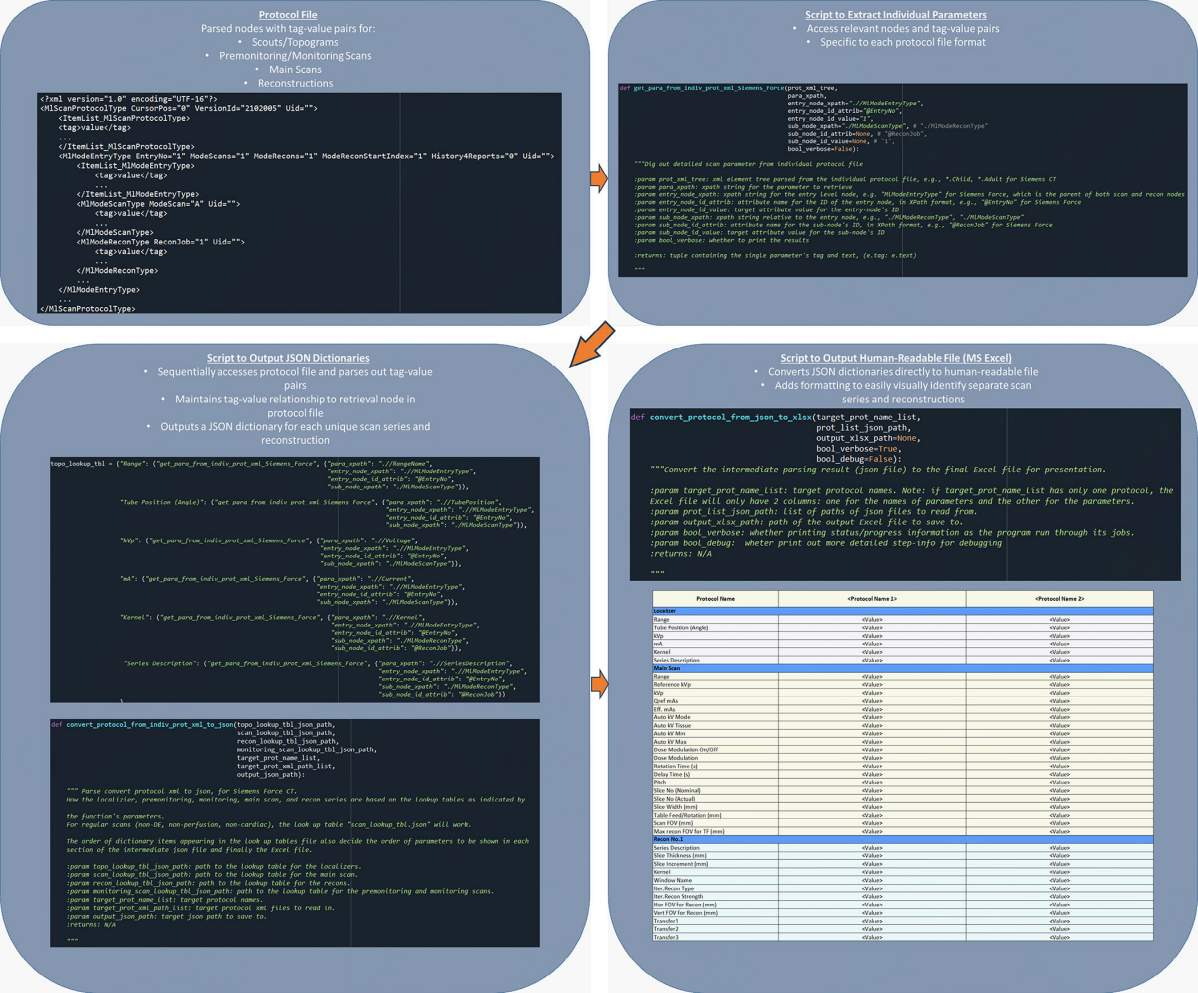
Flowchart with example Python scripts showing pseudo‐script of a Siemens Force protocol file, Python function for individual parameter extraction, Python code to generate JSON tables with tag‐value pairs for each series (scouts, main scans, etc.) and reconstruction from protocol files, and Python code to generate a user‐friendly Excel file for protocol review from protocol JSON tables.

JSON files were converted to a human‐readable, presentable format in Microsoft Excel. Note that any desired output format may be readily chosen. Excel was chosen due to its accessibility, convenient user‐interface, and community support with Python, including the Pandas toolbox.^26^ Individual scans were classified based on their series types and presented in a color‐coded manner for viewing. Our algorithm was developed to take one or multiple protocol files as inputs. If only one protocol file is specified, the parameters are output to an Excel file using the format specified by our algorithm, which was designed to easily delineate different series types (i.e., scout vs. monitoring vs. main scans). If multiple protocol files are input, our code outputs their parameters side‐by‐side in the same Excel file, and highlights differences between them in red, bold font in order to assist protocol review. Note that side‐by‐side protocol comparison is only possible for protocol files with the same protocol type (regular, cardiac, or dual‐energy) and that cover the same anatomy (head, torso, etc.), because the program tries to align scan and reconstruction series for the comparison. Figure 1 shows the entire sequence of our CT protocol extraction routine, including code snippets for each major step in the process.

## 3 RESULTS

**FIGURE 2:**
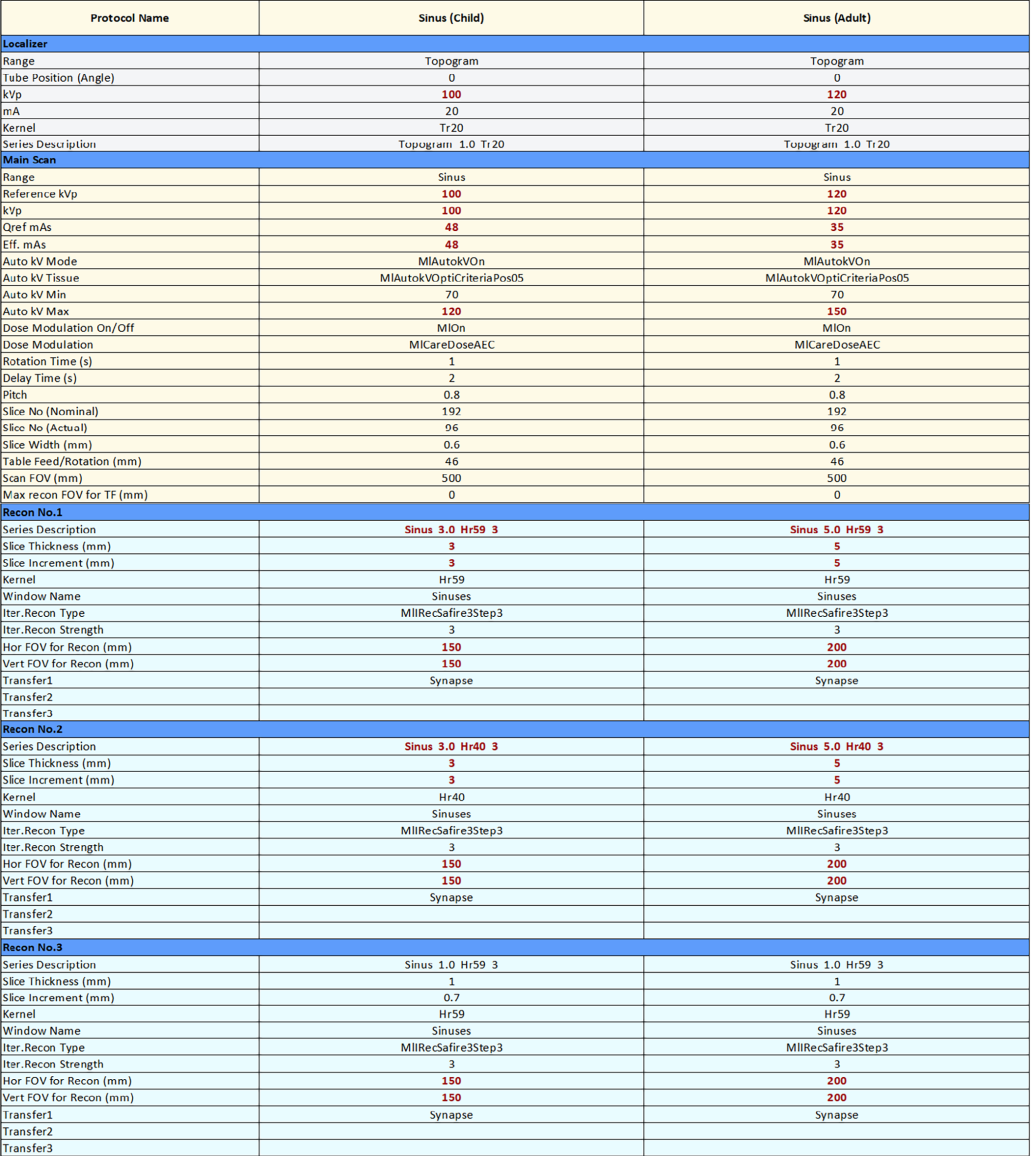
Example of two patient‐size based protocols converted from XML format to human‐readable (Excel) format for side‐by‐side comparison, for a Siemens Force scanner. Left column shows a pediatric Sinus protocol, and the right column shows an adult Sinus protocol. Differences in parameters are highlighted in bold red font.

Figures 2 and S1 show example cases for displaying similar protocols from Siemens Force and GE Optima 660, respectively, side‐by‐side for protocol review. Head protocols for pediatric and adult patients were chosen for Siemens Force, and chest protocols with different patient girth indications were chosen for GE Optima 660. Differences are automatically highlighted side‐by‐side in bold red font for easy identification. Parameters that differ between the displayed Siemens Force protocols in Figure 2 include kVp in both the scout and main scans, mAs in the main scan, and reconstruction kernel, slice thickness, and field‐of‐view (FOV) for the various main scan reconstructions In Figure S1, the main differences shown between the two GE Optima 660 protocols are the AEC settings, due to higher tube settings required for thicker patients, and reconstruction parameters including display FOV and iterative reconstruction strength.

Figures S2 and S3 show results from Siemens Force for protocol parameter extraction and display for cardiac and dual‐energy protocols, respectively. Figure S4 shows a protocol from Siemens Intevo Bold.

Several real‐life use case examples of our CT protocol parameter extraction methodology are highlighted in Figures S5–S8, which show several interrelated protocol review examples. A medical physicist at our facility deployed our algorithm for protocol review with the lead CT technologist and implemented various modifications to existing protocols. Figure S5 shows a pediatric neck protocol side‐by‐side with an adult neck protocol that served as the basis of its development. The pediatric neck protocol differs in terms of tube settings (kVp, mAs, rotation time), iterative reconstruction settings, number of reconstructions, and FOV. Figure S6 shows the same pediatric neck protocol from Figure S5 before and after edits were made during protocol review. Rotation time, which is a critical parameter for effective pediatric imaging, was made shorter, while one reconstruction series was removed entirely for optimization purposes, including decreasing exam processing time and ensuring consistent radiologist workflow. Figure S7 shows the same adult neck protocol from Figure S5 before and after edits were made. Changes were primarily made to reconstruction settings, including the addition of a high‐resolution reconstruction focused in the lungs. Figure S8 shows the adult neck protocol from Figures S5 and S7 side‐by‐side with an adult neck protocol of a higher dose class. While the scan parameters only differ by higher mAs settings and shorter delay time in one of the main scans for the higher dose class, there are many differences in reconstructions settings between the two protocols, including kernels, DICOM destinations, and number of reconstructions per main scan.

## 4 DISCUSSION

We have successfully developed and deployed an algorithm for extracting and displaying CT protocol parameters on scanners from different vendors, as well as various protocol types with different technical factors of interest. The program can extract and display any technical parameters and textual information of interest, which may greatly improve the comprehensiveness and depth of protocol review. These extracted parameters are not only related to the scanning and reconstruction settings, which are topics typically focused on during a physicist's review, but also parameters that are subtle yet impactful to the technologists' workflow and physician's image review process, such as DICOM destinations and ordering and naming of individual reconstructed series. While this present work only explores 3 different scanners from 2 different manufacturers, this methodology can be deployed on raw protocol files from any commercially available CT scanner; however, as previously discussed, adapting the code to extract parameters must be done individually for each scanner due to their unique formats.

Conversion from raw protocol file to a JSON dictionary of parameters needs to be handled on a per‐scanner basis due to unique raw protocol file formats, while conversion from JSON dictionary to a human‐readable format can be processed identically across all scanners. Raw protocol files have on the order of hundreds or, more typically, thousands of lines of code to parse through, whereas our algorithm distills this information into dozens of lines that are most relevant to technologists, radiologists and medical physicists for routine, streamlined review. Modifying which parameters are extracted and displayed can be done on the JSON dictionary‐level, thus separating this function from the backend code and enhancing the overall manageability of the code architecture. This was a key design feature that enabled us to deploy the frontend code (i.e., JSON to Excel conversion) we developed on all scanners within our fleet with minor modifications, while keeping the backend code (i.e., protocol file to JSON conversion) separate. As scanners are replaced or removed from a CT fleet over time, the frontend code could remain static (at the users’ discretion) while the backend code would only need to be modified on a per‐scanner basis, and only if a new protocol file format were introduced.

Our CT protocol parameter extraction tool has many real‐life use cases highlighted in this work: (1) comparing similar protocols side‐by‐side, such as the pediatric version and the adult version of protocols for the same indication (Figures 2 and S5), protocols covering the same anatomy but for different patient sizes (Figure S1), or similar protocols with different dose levels (Figure S8). Having key protocol parameters displayed side‐by‐side offered a visual guide for the physicist‐technologist team to review and adjust the technical parameters on the scanner. This eliminated the need to jump back and forth between protocols to compare parameters individually, reducing the potential for user error in this process. Similarly, this side‐by‐side display utility can be leveraged to develop protocols on a single scanner, and then apply them to another scanner, such as newer scanners from the same vendor. This can streamline the protocol development process on newer scanners, since all the parameters that need to be adjusted based on the pre‐existing protocols from other clinically deployed scanners will be highlighted for the user. (2) Comparison and confirmation of changes before and after editing a protocol—this tool will automatically highlight any changes made, which is helpful for reducing human errors and for record keeping. This functionality also assists when maintaining a database of version‐controlled protocol files before and after modifications were made (Figures S6 and S7), enabling users to easily track changes over time, especially with the feature we introduced to automatically highlight side‐by‐side differences in parameter values. This may also reduce user error by being able to easily spot unintended changes (Figure S6). (3) Uncovering inconsistencies in syntax across various protocols, which may help improve efficiency and reduce errors related to automation. For example, the second reconstruction for both protocols in Figure S1 are flagged as having different “Series Description” values, despite them alluding to the identical technical parameters: window range (i.e., anatomy of interest) and slice thickness. This is because one contains the syntax “MM” at the end, referring to the units of measurement for slice thickness (millimeters), whereas the other protocol doesn't. Although this minor inconsistency may not have a clear, immediate clinical impact, maintaining consistency in naming conventions is valuable, especially as radiology and informatics practices increasingly intersect.^27^, ^28^, ^29^, ^30^, ^31^ (4) Applications to other advanced imaging modalities, such as MRI and interventional fluoroscopy. Such modalities have similar need for protocol tracking, and our algorithm can sometimes be easily adapted to other modalities within the same vendor when the cross‐modality protocol files have similar structures and formats.

An additional feature that we did not explore, but could be readily implemented using our methodology, is comparing saved protocol parameters to actual performed protocols as denoted in DICOM headers, which would be an attractive feature for case‐by‐case dose tracking. While this would be a useful function for cataloging and flagging outlier exams, it would require 2 separate parameter extraction algorithms: one for the raw protocol file, and one for the DICOM file, which may contain proprietary fields unique to the vendor. However, DICOM follows similar logic to XML, making this a doable, albeit non‐trivial task to unite both algorithms under a common framework.

Edge cases need to be accounted for when extracting technical parameters from CT protocol files. Among the extracted tag‐value pairs, the tag names to be output to the human‐readable file, such as “kVp” can be directly specified and standardized in the JSON look‐up table, whereas the values, such as “120” (belonging to the tag “kVp”) are extracted as‐is from the raw protocol file, both due to how the JSON table is initialized. In many cases, numeric values are used in the protocol file to encode for more descriptive setting values that a technologist would see on the scanner's user interface. For example, the “Dose Modulation Submode”, an AEC setting on the GE Optima 660, can take on a value from 0 – 2 in the protocol file, which corresponds to no dose modulation (0), AutomA (1), and AutomA/SmartmA (2) on the user interface. These can be automatically decoded when presenting in the human‐readable format, but this may cause bugs if not all possible values are accounted for. Alternatively, a key may be inserted next to the value to indicate its meaning, which may be updated as new values become known. In either case, preparation must be taken to validate that the settings in the protocol file match what the user is seeing on the scanner's user interface.

One limiting factor in our methodology is the inability to perform cross‐vendor protocol comparisons, which is mainly due to the proprietary nature of CT scanners and protocols. For example, Siemens specifies “Effective mAs” and “Quality Reference mAs” to denote tube output in units of mAs, whereas GE has “Noise Index” and “Reference Noise Index”, which are in units of noise standard deviation. This is just one such example of incompatible parameter names across vendors, making cross‐comparison difficult and less meaningful. As a result, we elected to exclude this functionality in our code. We hope that more efforts will be made to standardize protocol parameters across vendors, such as through the Alliance for Quality Computed Tomography,^32^ as this would then enable meaningful cross‐vendor comparisons; however, that is outside the scope of this present study.

While CT protocol optimization generally focuses on technical exam parameters, such as tube, AEC and reconstruction settings, our protocol review algorithm includes non‐scan parameters that impact clinical workflow, including series descriptions and image transfer destinations. One capability of our algorithm is its ability to flag changes in reconstruction series ordering (Figures S7 and S8), which has significant impact for radiologists’ workflow. While non‐scan parameters may not traditionally fall under the medical physicists’ purview, they greatly impact the efficiency and smoothness of clinical workflow of technologists and physicians’ review process. Thus, including them in the routine protocol review process may increase overall efficiency. For example, uncovering unknown or unintended discrepancies between DICOM transfer destinations between similar protocols during routine protocol review would help identify and address common yet overlooked sources of inefficiency in the radiology IT chain.^33^, ^34^, ^35^, ^36^ In addition, having a hand on the informatics side of radiology is in line with the mission of Medical Physics 3.0 initiative to broaden the work scope and clinical impact of medical physicists.^37^

Our CT protocol parameter extraction tool may be deployed in a variety of ways for protocol review and management. Having a comprehensive, centralized database with version‐controlled CT protocols would greatly improve efficiency, especially at large medical enterprises where you would otherwise have to physically be present at each CT scanner to inspect and evaluate protocols.^6^ Wikis have become a popular format for maintaining large, centralized CT protocol databases.^10^, ^11^ Some of the many challenges associated with developing and maintaining these wikis are new CT scanners entering the fleet (with their own protocol file formats) and keeping the protocols in the wiki up‐to‐date relative to clinical practice. Our algorithm expedites these processes by decoupling the protocol file to wiki format conversion, as the MS Excel files can be embedded on a wiki system or stored or shared in an online workspace. In addition, our approach may also be deployed to automatically flag protocol deviations, which is a critical yet time‐consuming task during CT protocol review when done manually, through side‐by‐side protocol visualization and comparison.^38^

## 5 CONCLUSION

We have presented a user‐friendly tool for CT protocol parameter review tailored to the needs of medical physicists, radiologists, and technologists. Our CT protocol parameter extraction framework functions on an individual basis for both scanners and protocols, making it translatable across healthcare institutions of varying size and scope.

## AUTHOR CONTRIBUTIONS

Da Zhang designed the study and wrote the first draft of the code used for analysis. Andy LaBella edited the code throughout the study and wrote the first draft of the manuscript. Both authors obtained data and carried out data analysis. The manuscript was revised and edited based on input from both authors. Both authors approved the final version prior to publication.

## CONFLICT OF INTEREST STATEMENT

The authors declare no conflicts of interest.

## Supporting information

Supporting Information

## Data Availability

Source code for the Python scripts developed and used in this study are publicly available at https://github.com/alabella4/CT‐Protocol‐Management‐Tool/tree/main.
