# Supplementary material for: Protocol parameter extraction and centralization framework for comprehensive and in‐depth CT protocol review and management
Source: J Appl Clin Med Phys. 2024 Mar 11;25(4):e14316. doi: 10.1002/acm2.14316 (PMC11005989; doi:10.1002/acm2.14316)
Supplement: Supplementary file 1 — Supporting Information [file ACM2-25-e14316-s001.pdf]

Figure S1. Example of two patient age-based protocols converted from .proto format to human-readable (Excel) format for side-by-side comparison, for a GE Optima 660 scanner. Left column shows a pediatric chest protocol for patient girth 9 – 15 cm, and the right column shows a chest protocol for the patient girth 16 – 26 cm. Differences in parameters are highlighted in bold red font.

Protocol Name	chest_1.proto	chest_2.proto
**Localizer**		
Range	Scout	Scout
Tube Position (Angle)	90	90
kVp	**80**	**100**
mA	10	10
Series Description	**CHEST CLASS 1 9-15CM**	**CHEST CLASS 1 16-26CM**
**Localizer**		
Range	Scout	Scout
Tube Position (Angle)	0	0
kVp	**80**	**100**
mA	10	10
Series Description	**CHEST CLASS 1 9-15CM**	**CHEST CLASS 1 16-26CM**
**Main Scan**		
Range	**CHEST CLASS 1 9-15CM**	**CHEST CLASS1 16-26CM**
kVp	100	100
Auto kV Mode	kvModulationNone	kvModulationNone
Noise Index	**8.73**	**13.1**
Reference Noise Index	**8.73**	**13.1**
Auto mA minimum	**20**	**30**
Auto mA maximum	**150**	**115**
Dose Modulation On/Off	2	2
Dose Modulation Submode	2	2
Rotation Time (s)	0.5	0.5
Delay Time (s)	**0**	**3.4**
Pitch	1.3	1.3
Slice No (Nominal)	39	39
Slice Width (mm)	0.625	0.625
Table Feed/Rotation (mm)	55	55
Scan FOV (cm)	32	32
**Recon No. 1**		
Series Description	**CHEST CLASS 1 9-15CM**	**CHEST CLASS1 16-26CM**
Slice Thickness (mm)	5	5
Slice Increment (mm)	20	20
Kernel	Standard	Standard
Window Level	40	40
Window Width	400	400
Iter.Recon Type	AsirSlice	AsirSlice
Iter.Recon Strength	SS30:Slice	SS30:Slice
Display FOV for Recon (mm)	**20**	**25**
Transfer1	Synapse	Synapse
Transfer2	ACR	ACR
Transfer3		
**Recon No. 2**		
Series Description	**LUNG 2.5**	**LUNG 2.5MM**
Slice Thickness (mm)	2.5	2.5
Slice Increment (mm)	2.5	2.5
Kernel	Lung	Lung
Window Level	-500	-500
Window Width	1250	1250
Iter.Recon Type	AsirSlice	AsirSlice
Iter.Recon Strength	**SS20:Slice**	**SS30:Slice**
Display FOV for Recon (mm)	**20**	**25**
Transfer1	Synapse	Synapse
Transfer2	ACR	ACR
Transfer3		
**Recon No. 3**		
Series Description	**LUNG .635 X 3**	**LUNG 0.625MM**
Slice Thickness (mm)	0.625	0.625
Slice Increment (mm)	0.3	0.3
Kernel	Lung	Lung
Window Level	-500	-500
Window Width	1250	1250
Iter.Recon Type	AsirSlice	AsirSlice
Iter.Recon Strength	**SS30:Slice**	**SS20:Slice**
Display FOV for Recon (mm)	**20**	**25**
Transfer1	Synapse	Synapse
Transfer2	ACR	ACR
Transfer3		
**Recon No. 4**		
Series Description	STANDARD 0.625MM	STANDARD 0.625MM
Slice Thickness (mm)	0.625	0.625
Slice Increment (mm)	0.3	0.3
Kernel	Standard	Standard
Window Level	40	40
Window Width	400	400
Iter.Recon Type	AsirSlice	AsirSlice
Iter.Recon Strength	**SS30:Slice**	**SS20:Slice**
Display FOV for Recon (mm)	**20**	**25**
Transfer1	Synapse	Synapse
Transfer2	ACR	ACR
Transfer3		

Figure S2. Partial output Excel file for a gated cardiac CT protocol from Siemens Force. Output file is shown up to the first reconstruction of the first main scan. Not shown are several other reconstructions, as well as a second main scan and its reconstructions. The primary feature distinguishing cardiac protocols is bolus tracking, which is denoted as a separate series in the protocol file as well as the user interface for technologists.

Protocol Name	Gated TF Coronary Bolus
**Localizer**	
Range	Topogram LAT
Tube Position (Angle)	0
kVp	70
mA	20
Kernel	Tr20
Series Description	Topogram LAT 1.0 Tr20
**Localizer**	
Range	Topogram AP
Tube Position (Angle)	90
kVp	70
mA	20
Kernel	Tr20
Series Description	Topogram AP 1.0 Tr20
**PreMonitoring Scan**	
Range	PreMonitoring
Reference kVp	100
kVp	70
Qref mAs	288
mAs	20
Auto kV Mode	MlAutokVOff
Dose Modulation On/Off	MlOff
Rotation Time (s)	0.25
Delay Time (s)	2
Pitch	0
Slice No (Actual)	1
Slice Width (mm)	10
Scan FOV (mm)	500
No of Scans	1
**Contrast**	
Auto Trigger	true
Bolus Trigger Level	250
**Main Scan**	
Range	TF Diast 65%
Reference kVp	100
kVp	100
Qref mAs	288
Eff. mAs	288
Auto kV Mode	MlAutokVOn
Auto kV Tissue	MlAutokVOptiCriteriaPos11
Auto kV Min	70
Auto kV Max	100
Dose Modulation On/Off	MlOn
Dose Modulation	MlCareDoseAEC
Rotation Time (s)	0.25
Delay Time (s)	5
Pitch	3.2
Slice No (Nominal)	192
Slice No (Actual)	96
Slice Width (mm)	0.6
Table Feed/Rotation (mm)	184.3
Scan FOV (mm)	500
Max recon FOV for TF (mm)	0
**Recon No.1**	
Series Description	TF Diast 65% 3.0 Bv40 3 65%
Slice Thickness (mm)	3
Slice Increment (mm)	3
Kernel	Bv40
Window Name	Cardiac
Iter.Recon Type	MlIRecSafire3Step3
Iter.Recon Strength	3
Hor FOV for Recon (mm)	300
Vert FOV for Recon (mm)	300
Transfer1	Synapse
Transfer2	CardiacPACS
Transfer3	

Figure S3. Output Excel file for a dual-energy CT protocol from Siemens Force. The primary feature distinguishing dual-energy protocols is the multiple kVp and mAs settings within the main scan series.

**Protocol Name**	**DE Abdomen Seq**
**Localizer**	
Range	Topogram
Tube Position (Angle)	270
kVp	120
mA	20
Kernel	Tr20
Series Description	Topogram 1.0 Tr20
**Main Scan**	
Range	DE_AbdSeq
Reference kVp - A	100
kVp - A	100
Qref mAs - A	190
Eff. mAs - A	190
Reference kVp - B	150
kVp - B	150
Qref mAs - B	95
Eff. mAs - B	95
Auto kV Mode	MlAutokVOff
Auto kV Tissue	MlAutokVOptiCriteriaPos03
Auto kV Min	70
Auto kV Max	150
Dose Modulation On/Off	MlOn
Dose Modulation	MlCareDoseAEC
Rotation Time (s)	0.5
Delay Time (s)	2
Pitch	0
Slice No (Nominal)	128
Slice No (Actual)	64
Slice Width (mm)	0.6
Table Feed/Rotation (mm)	15
Scan FOV (mm)	500
Max recon FOV for TF (mm)	0
**Recon No.1**	
Series Description	DE_AbdSeq 5.0 Br40 3
Slice Thickness (mm)	5
Slice Increment (mm)	5
Kernel	Br40
Window Name	Abdomen
Iter.Recon Type	MlIRecSafire3Step3
Iter.Recon Strength	3
Hor FOV for Recon (mm)	300
Vert FOV for Recon (mm)	300
Transfer1	Synapse
Transfer2	
Transfer3	

Figure S4. Output Excel file for a pediatric abdomen CT protocol from Siemens Intevo Bold.

**Protocol Name**	**ABDOMEN_UNDER_55KG_CLASS_1_R.MlChild**
**Localizer**	
Range	Topogram
Tube Position	MlTubePosLateral
kVp	110
mA	25
Kernel	T20f standard
Series Description	Topogram 0.6 T20f
**Localizer**	
Range	Topogram
Tube Position	MlTubePosPa
kVp	110
mA	25
Kernel	T20f standard
Series Description	Topogram 0.6 T20f
**Main Scan**	
Range	AbdRoutine
Reference kVp	110
kVp	110
Qref mAs	150
Eff mAs	150
Auto kV Mode	MlOff
Dose Modulation On/Off	MlAutomaticExposureControl
Dose Modulation	MlAutomaticExposureControl
Rotation Time (s)	0.6
Delay Time (s)	3
Pitch	1.3
Slice No (Nominal)	16
Slice No (Actual)	16
Slice Width (mm)	0.6
Table Feed/Rotation (mm)	12.5
Scan FOV (mm)	0
**Recon No. 1**	
Series Description	AbdRoutine 5.0 B41s
Slice Thickness	5
Slice Increment	5
Kernel	B41s medium +
Window Name	Baby Abdomen
Iter. Recon Strength	MlPrimary
Hor FOV for Recon (mm)	180
Vert FOV for Recon (mm)	180
Transfer1	
Transfer2	
Transfer3	
**Recon No. 2**	
Series Description	AbdRoutine 0.75 B41s
Slice Thickness	0.75
Slice Increment	0.75
Kernel	B41s medium +
Window Name	Baby Abdomen
Iter. Recon Strength	MlPrimary
Hor FOV for Recon (mm)	180
Vert FOV for Recon (mm)	180
Transfer1	
Transfer2	
Transfer3	

Figure S5. Output Excel file from Siemens Force comparing the differences between adult and pediatric neck protocols for the same indications. The empty rows for "Recon No. 3" of the 1st scan series of the adult protocol indicates different number of reconstructions between the adult and the pediatric protocols.

Protocol Name	TF_NECK_CAP_UNDER_55KG__CLASS_2Customized (Child)	TF_NECK_CAP_OVER_55KG_CLASS_2 (Adult)
**Localizer**		
Range	Topogram	Topogram
Tube Position (Angle)	**90**	**0**
kVp	**100**	**120**
mA	20	20
Kernel	Tr20	Tr20
Series Description	Topogram 1.0 Tr20	Topogram 1.0 Tr20
**Localizer**		
Range	Topogram	Topogram
Tube Position (Angle)	**0**	**90**
kVp	**100**	**120**
mA	20	20
Kernel	Tr20	Tr20
Series Description	Topogram 1.0 Tr20	Topogram 1.0 Tr20
**Main Scan**		
Range	Neck	Neck
Reference kVp	**100**	**120**
kVp	**100**	**120**
Qref mAs	**163**	**116**
Eff. mAs	**163**	**116**
Auto kV Mode	MlAutokVOn	MlAutokVOn
Auto kV Tissue	MlAutokVOptiCriteriaPos07	MlAutokVOptiCriteriaPos07
Auto kV Min	**70**	**80**
Auto kV Max	**120**	**150**
Dose Modulation On/Off	MlOn	MlOn
Dose Modulation	MlCareDoseAEC	MlCareDoseAEC
Rotation Time (s)	**1**	**0.25**
Delay Time (s)	2	2
Pitch	0.8	0.8
Slice No (Nominal)	192	192
Slice No (Actual)	96	96
Slice Width (mm)	0.6	0.6
Table Feed/Rotation (mm)	46	46
Scan FOV (mm)	500	500
Max recon FOV for TF (mm)	0	0
**Recon No.1**		
Series Description	**Neck 3.0 Br40 2**	**Neck 3.0 Br44 3**
Slice Thickness (mm)	3	3
Slice Increment (mm)	3	3
Kernel	**Br40**	**Br44**
Window Name	**Baby Neck**	**Mediastinum**
Iter.Recon Type	MlIRecSafire3Step3	MlIRecSafire3Step3
Iter.Recon Strength	**2**	**3**
Hor FOV for Recon (mm)	**120**	**250**
Vert FOV for Recon (mm)	**120**	**250**
Transfer1	Synapse	Synapse
Transfer2		
Transfer3		
**Recon No.2**		
Series Description	**Neck 0.6 Br44 2**	**THIN SOFT**
Slice Thickness (mm)	0.6	0.6
Slice Increment (mm)	0.3	0.3
Kernel	Br44	Br44
Window Name	**Baby Neck**	**Mediastinum**
Iter.Recon Type	MlIRecSafire3Step3	MlIRecSafire3Step3
Iter.Recon Strength	**2**	**3**
Hor FOV for Recon (mm)	**120**	**250**
Vert FOV for Recon (mm)	**120**	**250**
Transfer1	Synapse	Synapse
Transfer2		
Transfer3		
**Recon No.3**		
Series Description	**Neck 0.6 Br69**	
Slice Thickness (mm)	**0.6**	
Slice Increment (mm)	**0.3**	
Kernel	**Br69**	
Window Name	**Bone**	
Iter.Recon Type	**MlIRecOff**	
Iter.Recon Strength	0	
Hor FOV for Recon (mm)	**120**	
Vert FOV for Recon (mm)	**120**	
Transfer1	**Synapse**	
Transfer2		
Transfer3		

Main Scan		
Range	**TF CHEST ABD**	**TF CAP >55kg**
Reference kVp	120	120
kVp	**100**	**110**
Qref mAs	100	100
Eff. mAs	**136**	**114**
Auto kV Mode	MlAutokVOn	MlAutokVOn
Auto kV Tissue	MlAutokVOptiCriteriaPos07	MlAutokVOptiCriteriaPos07
Auto kV Min	80	80
Auto kV Max	120	120
Dose Modulation On/Off	MlOn	MlOn
Dose Modulation	MlCareDoseAEC	MlCareDoseAEC
Rotation Time (s)	0.25	0.25
Delay Time (s)	**36**	**15**
Pitch	1.9	1.9
Slice No (Nominal)	192	192
Slice No (Actual)	96	96
Slice Width (mm)	0.6	0.6
Table Feed/Rotation (mm)	109.4	109.4
Scan FOV (mm)	500	500
Max recon FOV for TF (mm)	469	469
**Recon No.1**		
Series Description	**TF CHEST ABD CLASS 1 5.0 Br44 3**	**TF CAP >55kg 5.0 Br44 3**
Slice Thickness (mm)	5	5
Slice Increment (mm)	5	5
Kernel	Br44	Br44
Window Name	**Baby Abdomen**	**Mediastinum**
Iter.Recon Type	MlIRecSafire3Step3	MlIRecSafire3Step3
Iter.Recon Strength	3	3
Hor FOV for Recon (mm)	**180**	**300**
Vert FOV for Recon (mm)	**180**	**300**
Transfer1	Synapse	Synapse
Transfer2		
Transfer3		
**Recon No.2**		
Series Description	**TF CHEST ABD 2.0 Bl64 3**	**LUNG >55kg 2.0 Bl64 3**
Slice Thickness (mm)	2	2
Slice Increment (mm)	2	2
Kernel	Bl64	Bl64
Window Name	**Baby Lung**	**Lung**
Iter.Recon Type	MlIRecSafire3Step3	MlIRecSafire3Step3
Iter.Recon Strength	3	3
Hor FOV for Recon (mm)	**180**	**300**
Vert FOV for Recon (mm)	**180**	**300**
Transfer1	Synapse	Synapse
Transfer2		
Transfer3		
**Recon No.3**		
Series Description	**STD THINS**	**THIN LUNG**
Slice Thickness (mm)	0.6	0.6
Slice Increment (mm)	0.3	0.3
Kernel	**Br44**	**Bl64**
Window Name	**Mediastinum**	**Lung**
Iter.Recon Type	MlIRecSafire3Step3	MlIRecSafire3Step3
Iter.Recon Strength	3	3
Hor FOV for Recon (mm)	**180**	**300**
Vert FOV for Recon (mm)	**180**	**300**
Transfer1		**Synapse**
Transfer2		
Transfer3		
**Recon No.4**		
Series Description	**LUNG THINS**	**THIN STANDARD**
Slice Thickness (mm)	0.6	0.6
Slice Increment (mm)	0.3	0.3
Kernel	**Bl64**	**Br44**
Window Name	**Baby Lung**	**Mediastinum**
Iter.Recon Type	MlIRecSafire3Step3	MlIRecSafire3Step3
Iter.Recon Strength	3	3
Hor FOV for Recon (mm)	**180**	**300**
Vert FOV for Recon (mm)	**180**	**300**
Transfer1	**Synapse**	
Transfer2		
Transfer3		

Figure S6. Output Excel file from Siemens Force displaying pediatric neck protocol before and after edits were made via annual protocol review between medical physicists and CT technologists. The empty rows for “Recon No. 3” of the 1st scan series of the revised protocol (right column) indicates a reconstruction being removed.

Protocol Name	TF_NECK_CAP_Class-2_Under_55KG (Before)	TF_NECK_CAP_Class-2_Under_55KG (After)
**Localizer**		
Range	Topogram	Topogram
Tube Position (Angle)	90	90
kVp	100	100
mA	20	20
Kernel	Tr20	Tr20
Series Description	Topogram 1.0 Tr20	Topogram 1.0 Tr20
**Localizer**		
Range	Topogram	Topogram
Tube Position (Angle)	0	0
kVp	100	100
mA	20	20
Kernel	Tr20	Tr20
Series Description	Topogram 1.0 Tr20	Topogram 1.0 Tr20
**Main Scan**		
Range	Neck	Neck
Reference kVp	100	100
kVp	100	100
Qref mAs	163	163
Eff. mAs	163	163
Auto kV Mode	MlAutokVOn	MlAutokVOn
Auto kV Tissue	MlAutokVOptiCriteriaPos07	MlAutokVOptiCriteriaPos07
Auto kV Min	70	70
Auto kV Max	120	120
Dose Modulation On/Off	MlOn	MlOn
Dose Modulation	MlCareDoseAEC	MlCareDoseAEC
Rotation Time (s)	**1**	**0.25**
Delay Time (s)	2	2
Pitch	0.8	0.8
Slice No (Nominal)	192	192
Slice No (Actual)	96	96
Slice Width (mm)	0.6	0.6
Table Feed/Rotation (mm)	46	46
Scan FOV (mm)	500	500
Max recon FOV for TF (mm)	0	0
**Recon No.1**		
Series Description	**Neck 3.0 Br40 2**	**Neck 3.0 Br40 3**
Slice Thickness (mm)	3	3
Slice Increment (mm)	3	3
Kernel	Br40	Br40
Window Name	Baby Neck	Baby Neck
Iter.Recon Type	MlIRecSafire3Step3	MlIRecSafire3Step3
Iter.Recon Strength	**2**	**3**
Hor FOV for Recon (mm)	120	120
Vert FOV for Recon (mm)	120	120
Transfer1	Synapse	Synapse
Transfer2		
Transfer3		
**Recon No.2**		
Series Description	**Neck 0.6 Br44 2**	**THIN STD**
Slice Thickness (mm)	0.6	0.6
Slice Increment (mm)	0.3	0.3
Kernel	**Br44**	**Br40**
Window Name	Baby Neck	Baby Neck
Iter.Recon Type	MlIRecSafire3Step3	MlIRecSafire3Step3
Iter.Recon Strength	**2**	**3**
Hor FOV for Recon (mm)	120	120
Vert FOV for Recon (mm)	120	120
Transfer1	Synapse	Synapse
Transfer2		
Transfer3		
**Recon No.3**		
Series Description	**Neck 0.6 Br69**	
Slice Thickness (mm)	**0.6**	
Slice Increment (mm)	**0.3**	
Kernel	**Br69**	
Window Name	**Bone**	
Iter.Recon Type	**MlIRecOff**	
Iter.Recon Strength	0	
Hor FOV for Recon (mm)	**120**	
Vert FOV for Recon (mm)	**120**	
Transfer1	**Synapse**	
Transfer2		
Transfer3		

**Main Scan**		
Range	TF CHEST ABD	TF CHEST ABD
Reference kVp	120	120
kVp	**100**	**120**
Qref mAs	100	100
Eff. mAs	**136**	**100**
Auto kV Mode	MlAutokVOn	MlAutokVOn
Auto kV Tissue	MlAutokVOptiCriteriaPos07	MlAutokVOptiCriteriaPos07
Auto kV Min	80	80
Auto kV Max	120	120
Dose Modulation On/Off	MlOn	MlOn
Dose Modulation	MlCareDoseAEC	MlCareDoseAEC
Rotation Time (s)	0.25	0.25
Delay Time (s)	**36**	**6**
Pitch	1.9	1.9
Slice No (Nominal)	192	192
Slice No (Actual)	96	96
Slice Width (mm)	0.6	0.6
Table Feed/Rotation (mm)	109.4	109.4
Scan FOV (mm)	500	500
Max recon FOV for TF (mm)	469	469
**Recon No.1**		
Series Description	**TF CHEST ABD CLASS 1 5.0 Br44 3**	**TF C/A/P <55KG CLASS 2 5.0 Br44 3**
Slice Thickness (mm)	5	5
Slice Increment (mm)	5	5
Kernel	Br44	Br44
Window Name	**Baby Abdomen**	**Mediastinum**
Iter.Recon Type	MlIRecSafire3Step3	MlIRecSafire3Step3
Iter.Recon Strength	3	3
Hor FOV for Recon (mm)	180	180
Vert FOV for Recon (mm)	180	180
Transfer1	Synapse	Synapse
Transfer2		
Transfer3		
**Recon No.2**		
Series Description	**TF CHEST ABD 2.0 Bl64 3**	**2X2 LUNG**
Slice Thickness (mm)	2	2
Slice Increment (mm)	2	2
Kernel	Bl64	Bl64
Window Name	Baby Lung	Baby Lung
Iter.Recon Type	MlIRecSafire3Step3	MlIRecSafire3Step3
Iter.Recon Strength	3	3
Hor FOV for Recon (mm)	180	180
Vert FOV for Recon (mm)	180	180
Transfer1	Synapse	Synapse
Transfer2		
Transfer3		
**Recon No.3**		
Series Description	**STD THINS**	**THIN STD**
Slice Thickness (mm)	0.6	0.6
Slice Increment (mm)	0.3	0.3
Kernel	Br44	Br44
Window Name	Mediastinum	Mediastinum
Iter.Recon Type	MlIRecSafire3Step3	MlIRecSafire3Step3
Iter.Recon Strength	3	3
Hor FOV for Recon (mm)	180	180
Vert FOV for Recon (mm)	180	180
Transfer1		**Synapse**
Transfer2		
Transfer3		
**Recon No.4**		
Series Description	**LUNG THINS**	**THIN LUNG**
Slice Thickness (mm)	0.6	0.6
Slice Increment (mm)	0.3	0.3
Kernel	Bl64	Bl64
Window Name	Baby Lung	Baby Lung
Iter.Recon Type	MlIRecSafire3Step3	MlIRecSafire3Step3
Iter.Recon Strength	3	3
Hor FOV for Recon (mm)	180	180
Vert FOV for Recon (mm)	180	180
Transfer1	Synapse	Synapse
Transfer2		
Transfer3		

Figure S7. Output Excel file from Siemens Force displaying adult neck protocol before and after edits were made via annual protocol review between medical physicists and CT technologists. Note the change of order and number of reconstruction series originated from the 2nd scan series ("CAP").

Protocol Name	TF_NECK_CAP_OVER_55KG_CLASS_2 (Adult)	TF_NECK_CAP_OVER_55KG_CLASS_2 (Adult)
**Localizer**		
Range	Topogram	Topogram
Tube Position (Angle)	**0**	**90**
kVp	120	120
mA	20	20
Kernel	Tr20	Tr20
Series Description	Topogram 1.0 Tr20	Topogram 1.0 Tr20
**Localizer**		
Range	Topogram	Topogram
Tube Position (Angle)	**90**	**0**
kVp	120	120
mA	20	20
Kernel	Tr20	Tr20
Series Description	Topogram 1.0 Tr20	Topogram 1.0 Tr20
**Main Scan**		
Range	Neck	Neck
Reference kVp	120	120
kVp	120	120
Qref mAs	116	116
Eff. mAs	116	116
Auto kV Mode	MlAutokVOn	MlAutokVOn
Auto kV Tissue	MlAutokVOptiCriteriaPos07	MlAutokVOptiCriteriaPos07
Auto kV Min	80	80
Auto kV Max	150	150
Dose Modulation On/Off	MlOn	MlOn
Dose Modulation	MlCareDoseAEC	MlCareDoseAEC
Rotation Time (s)	0.25	0.25
Delay Time (s)	2	2
Pitch	0.8	0.8
Slice No (Nominal)	192	192
Slice No (Actual)	96	96
Slice Width (mm)	0.6	0.6
Table Feed/Rotation (mm)	46	46
Scan FOV (mm)	500	500
Max recon FOV for TF (mm)	0	0
**Recon No.1**		
Series Description	Neck 3.0 Br44 3	Neck 3.0 Br44 3
Slice Thickness (mm)	3	3
Slice Increment (mm)	3	3
Kernel	Br44	Br44
Window Name	Mediastinum	Mediastinum
Iter.Recon Type	MlIRecSafire3Step3	MlIRecSafire3Step3
Iter.Recon Strength	3	3
Hor FOV for Recon (mm)	250	250
Vert FOV for Recon (mm)	250	250
Transfer1	Synapse	Synapse
Transfer2		
Transfer3		
**Recon No.2**		
Series Description	THIN SOFT	THIN SOFT
Slice Thickness (mm)	0.6	0.6
Slice Increment (mm)	0.3	0.3
Kernel	Br44	Br44
Window Name	Mediastinum	Mediastinum
Iter.Recon Type	MlIRecSafire3Step3	MlIRecSafire3Step3
Iter.Recon Strength	3	3
Hor FOV for Recon (mm)	250	250
Vert FOV for Recon (mm)	250	250
Transfer1	Synapse	Synapse
Transfer2		
Transfer3		

**Main Scan**		
Range	TF CAP >55kg	TF CAP >55kg
Reference kVp	120	120
kVp	**110**	**120**
Qref mAs	100	100
Eff. mAs	**114**	**100**
Auto kV Mode	MlAutokVOn	MlAutokVOn
Auto kV Tissue	MlAutokVOptiCriteriaPos07	MlAutokVOptiCriteriaPos07
Auto kV Min	80	80
Auto kV Max	120	120
Dose Modulation On/Off	MlOn	MlOn
Dose Modulation	MlCareDoseAEC	MlCareDoseAEC
Rotation Time (s)	0.25	0.25
Delay Time (s)	15	15
Pitch	1.9	1.9
Slice No (Nominal)	192	192
Slice No (Actual)	96	96
Slice Width (mm)	0.6	0.6
Table Feed/Rotation (mm)	109.4	109.4
Scan FOV (mm)	500	500
Max recon FOV for TF (mm)	469	469
**Recon No.1**		
Series Description	TF CAP >55kg 5.0 Br44 3	TF CAP >55kg 5.0 Br44 3
Slice Thickness (mm)	5	5
Slice Increment (mm)	5	5
Kernel	Br44	Br44
Window Name	Mediastinum	Mediastinum
Iter.Recon Type	MlIRecSafire3Step3	MlIRecSafire3Step3
Iter.Recon Strength	3	3
Hor FOV for Recon (mm)	300	300
Vert FOV for Recon (mm)	300	300
Transfer1	Synapse	Synapse
Transfer2		
Transfer3		
**Recon No.2**		
Series Description	**LUNG >55kg 2.0 Bl64 3**	**2X2 LUNG**
Slice Thickness (mm)	2	2
Slice Increment (mm)	2	2
Kernel	Bl64	Bl64
Window Name	Lung	Lung
Iter.Recon Type	MlIRecSafire3Step3	MlIRecSafire3Step3
Iter.Recon Strength	3	3
Hor FOV for Recon (mm)	300	300
Vert FOV for Recon (mm)	300	300
Transfer1	Synapse	Synapse
Transfer2		
Transfer3		
**Recon No.3**		
Series Description	**THIN LUNG**	**THIN STD**
Slice Thickness (mm)	0.6	0.6
Slice Increment (mm)	0.3	0.3
Kernel	**Bl64**	**Br44**
Window Name	**Lung**	**Mediastinum**
Iter.Recon Type	MlIRecSafire3Step3	MlIRecSafire3Step3
Iter.Recon Strength	3	3
Hor FOV for Recon (mm)	300	300
Vert FOV for Recon (mm)	300	300
Transfer1	Synapse	Synapse
Transfer2		
Transfer3		
**Recon No.4**		
Series Description	THIN STANDARD	THIN STANDARD
Slice Thickness (mm)	0.6	0.6
Slice Increment (mm)	0.3	0.3
Kernel	Br44	Br44
Window Name	Mediastinum	Mediastinum
Iter.Recon Type	MlIRecSafire3Step3	MlIRecSafire3Step3
Iter.Recon Strength	3	3
Hor FOV for Recon (mm)	300	300
Vert FOV for Recon (mm)	300	300
Transfer1		
Transfer2		
Transfer3		
**Recon No.5**		
Series Description		**THIN LUNG**
Slice Thickness (mm)		**0.6**
Slice Increment (mm)		**0.3**
Kernel		**Bl64**
Window Name		**Lung**
Iter.Recon Type		**MlIRecSafire3Step3**
Iter.Recon Strength		**3**
Hor FOV for Recon (mm)		**300**
Vert FOV for Recon (mm)		**300**
Transfer1		**Synapse**
Transfer2		
Transfer3		

Figure S8. Output Excel file from Siemens Force displaying adult neck protocols with different dose classes (Class 1 = normal dose, Class 2 = low dose). Note the difference in the order and number of reconstruction series originated from the 2nd scan (CAP).

Protocol Name	TF_NECK_CAP_OVER_55KG_CLASS_1 (Adult)	TF_NECK_CAP_OVER_55KG_CLASS_2 (Adult)
**Localizer**		
Range	Topogram	Topogram
Tube Position (Angle)	90	90
kVp	120	120
mA	20	20
Kernel	Tr20	Tr20
Series Description	Topogram 1.0 Tr20	Topogram 1.0 Tr20
**Localizer**		
Range	Topogram	Topogram
Tube Position (Angle)	0	0
kVp	120	120
mA	20	20
Kernel	Tr20	Tr20
Series Description	Topogram 1.0 Tr20	Topogram 1.0 Tr20
**Main Scan**		
Range	Neck	Neck
Reference kVp	120	120
kVp	120	120
Qref mAs	116	116
Eff. mAs	116	116
Auto kV Mode	MlAutokVOn	MlAutokVOn
Auto kV Tissue	MlAutokVOptiCriteriaPos07	MlAutokVOptiCriteriaPos07
Auto kV Min	80	80
Auto kV Max	150	150
Dose Modulation On/Off	MlOn	MlOn
Dose Modulation	MlCareDoseAEC	MlCareDoseAEC
Rotation Time (s)	0.25	0.25
Delay Time (s)	2	2
Pitch	0.8	0.8
Slice No (Nominal)	192	192
Slice No (Actual)	96	96
Slice Width (mm)	0.6	0.6
Table Feed/Rotation (mm)	46	46
Scan FOV (mm)	500	500
Max recon FOV for TF (mm)	0	0
**Recon No.1**		
Series Description	**Neck 3.0 Br40 3**	**Neck 3.0 Br44 3**
Slice Thickness (mm)	3	3
Slice Increment (mm)	3	3
Kernel	**Br40**	**Br44**
Window Name	Mediastinum	Mediastinum
Iter.Recon Type	MlIRecSafire3Step3	MlIRecSafire3Step3
Iter.Recon Strength	3	3
Hor FOV for Recon (mm)	250	250
Vert FOV for Recon (mm)	250	250
Transfer1	Synapse	Synapse
Transfer2		
Transfer3		
**Recon No.2**		
Series Description	THIN SOFT	THIN SOFT
Slice Thickness (mm)	0.6	0.6
Slice Increment (mm)	0.3	0.3
Kernel	**Br40**	**Br44**
Window Name	Mediastinum	Mediastinum
Iter.Recon Type	MlIRecSafire3Step3	MlIRecSafire3Step3
Iter.Recon Strength	3	3
Hor FOV for Recon (mm)	250	250
Vert FOV for Recon (mm)	250	250
Transfer1	Synapse	Synapse
Transfer2		
Transfer3		

**Main Scan**		
Range	TF CAP >55kg	TF CAP >55kg
Reference kVp	120	120
kVp	120	120
Qref mAs	**150**	**100**
Eff. mAs	**150**	**100**
Auto kV Mode	MlAutokVOn	MlAutokVOn
Auto kV Tissue	MlAutokVOptiCriteriaPos07	MlAutokVOptiCriteriaPos07
Auto kV Min	80	80
Auto kV Max	120	120
Dose Modulation On/Off	MlOn	MlOn
Dose Modulation	MlCareDoseAEC	MlCareDoseAEC
Rotation Time (s)	0.25	0.25
Delay Time (s)	**8**	**15**
Pitch	1.9	1.9
Slice No (Nominal)	192	192
Slice No (Actual)	96	96
Slice Width (mm)	0.6	0.6
Table Feed/Rotation (mm)	109.4	109.4
Scan FOV (mm)	500	500
Max recon FOV for TF (mm)	469	469
**Recon No.1**		
Series Description	TF CAP >55kg 5.0 Br44 3	TF CAP >55kg 5.0 Br44 3
Slice Thickness (mm)	5	5
Slice Increment (mm)	5	5
Kernel	Br44	Br44
Window Name	Mediastinum	Mediastinum
Iter.Recon Type	MlIRecSafire3Step3	MlIRecSafire3Step3
Iter.Recon Strength	3	3
Hor FOV for Recon (mm)	300	300
Vert FOV for Recon (mm)	300	300
Transfer1	Synapse	Synapse
Transfer2		
Transfer3		
**Recon No.2**		
Series Description	2X2 LUNG	2X2 LUNG
Slice Thickness (mm)	2	2
Slice Increment (mm)	2	2
Kernel	Bl64	Bl64
Window Name	Lung	Lung
Iter.Recon Type	MlIRecSafire3Step3	MlIRecSafire3Step3
Iter.Recon Strength	3	3
Hor FOV for Recon (mm)	300	300
Vert FOV for Recon (mm)	300	300
Transfer1	Synapse	Synapse
Transfer2		
Transfer3		
**Recon No.3**		
Series Description	THIN STD	THIN STD
Slice Thickness (mm)	0.6	0.6
Slice Increment (mm)	0.3	0.3
Kernel	Br44	Br44
Window Name	Mediastinum	Mediastinum
Iter.Recon Type	MlIRecSafire3Step3	MlIRecSafire3Step3
Iter.Recon Strength	3	3
Hor FOV for Recon (mm)	300	300
Vert FOV for Recon (mm)	300	300
Transfer1	Synapse	Synapse
Transfer2		
Transfer3		
**Recon No.4**		
Series Description	**THIN LUNG**	**THIN STANDARD**
Slice Thickness (mm)	0.6	0.6
Slice Increment (mm)	0.3	0.3
Kernel	**Bl64**	**Br44**
Window Name	**Lung**	**Mediastinum**
Iter.Recon Type	MlIRecSafire3Step3	MlIRecSafire3Step3
Iter.Recon Strength	3	3
Hor FOV for Recon (mm)	300	300
Vert FOV for Recon (mm)	300	300
Transfer1	**Synapse**	
Transfer2		
Transfer3		
**Recon No.5**		
Series Description		**THIN LUNG**
Slice Thickness (mm)		**0.6**
Slice Increment (mm)		**0.3**
Kernel		**Bl64**
Window Name		**Lung**
Iter.Recon Type		**MlIRecSafire3Step3**
Iter.Recon Strength		**3**
Hor FOV for Recon (mm)		**300**
Vert FOV for Recon (mm)		**300**
Transfer1		**Synapse**
Transfer2		
Transfer3		
